# Testing for racial bias using inconsistent perceptions of race

**DOI:** 10.1126/sciadv.adx5829

**Published:** 2025-11-19

**Authors:** Nora Gera, Emma Pierson

**Affiliations:** ^1^ Department of Computer Science, Cornell University, Ithaca, NY, USA.; ^2^ Department of Electrical Engineering and Computer Sciences, University of California, Berkeley, CA, USA.

## Abstract

Tests for racial bias commonly assess whether two people of different races are treated differently. A fundamental challenge is that, because two people may differ in many ways, factors besides race might explain differences in treatment. Here, we propose a test for bias that circumvents the difficulty of comparing two people by instead assessing whether the same person is treated differently when their race is perceived differently. We apply our method to test for bias in police traffic stops, finding that the same driver is likelier to be searched or arrested by police when they are perceived as Hispanic than when they are perceived as white. Our test is broadly applicable to other datasets where race, gender, or other identity data are perceived rather than self-reported, and the same person is observed multiple times.

## Teaser

A proposed test for racial bias assesses whether the same person is treated differently when their race is perceived differently.

## INTRODUCTION

Racial bias contributes to inequities in high-stakes settings including hiring, criminal justice, health care, and housing (*1*–*6*). However, rigorously quantifying bias is challenging for a fundamental reason: If two people of different races are treated differently, it is difficult to know whether this is because of their race or because of other confounding factors, an example of the omitted variable bias problem (*3*, *4*, *7*–*10*). To circumvent this issue, a long literature on quantifying racial bias has used a host of strategies, including randomized controlled experiments (audit studies), natural experiments, and controlling for relevant factors (*2*, *11*–*17*). The common goal of these approaches is to estimate disparities in treatment across race groups after removing or adjusting for confounds that differ between groups. Remaining differences in how two people of different races are treated can then be attributed to bias.

Here, we take a fundamentally different approach. Instead of comparing two people of different races, we study the same person over time. At first glance, this would seemingly remove the variation needed to measure bias, since past work has often treated a person’s race as fixed over time (*18*). However, research has shown that perceptions of a single person’s race can be inconsistent and fluid over time (*19*–*23*). According to constructivist theories of race, observers rely on context clues—e.g., the clothing someone is wearing (*24*)—to perceive a person’s race. A long line of research attests to the complex, contextual psychological processes underlying the perception of racial and other social categories (*25*–*30*). Perceived race can differ from a person’s self-identified race and can change depending on the observer and other aspects of the situation (*18*, *19*, *31*–*39*).

Our core approach is to use this inconsistency in perceived race to test for a specific type of racial bias: Is the same person treated differently when their race is perceived differently? For example, if the same person receives worse treatment when they are perceived as Hispanic than when they are perceived as white, then this suggests anti-Hispanic bias. We motivate our test by building on a constructivist model of discrimination (*18*), as we discuss in more detail below.

Our work relates most closely to two important lines of literature. First, we build on past work that studies changes in perceived race over time (*20*–*23*, *40*–*42*). This literature documents the many ways in which perceptions of race reflect societal patterns of bias and inequality—for example, Noghanibehambari and Fletcher (*23*) study families in which one sibling passes for white and the other does not, finding that the passing siblings live longer; Cornwell *et al.* (*42*) finds that workers in Brazil receive higher wages from employers who perceive them as white. Our work builds on this literature by proposing a simple, general test for bias in a downstream human decision and applying this test to assess bias in police search decisions. Second, our work builds on the literature that compares perceived to self-identified race. The focus of this literature differs from our own goal of proposing a general test for bias in a downstream human decision. Instead, this literature typically assesses how often perceived and self-identified race differ; the factors that predict this will occur (which often reflect societal inequalities and biases) (*31*–*33*), and how discrepancies between perceived and self-identified race may affect measurements of disparities (*32*, *34*, *35*, *39*). This literature also differs from our own work because it measures discrepancies between perceived race and self-identified race; in contrast, we measure discrepancies in perceived race over time and do not require data on self-identified race, which is often unavailable.

We illustrate our method by applying it to test for racial bias in police traffic stops using data from three states—Arizona, Colorado, and Texas—which provide the data necessary to track the same driver across multiple stops. Pooling data across all three states, 9% of drivers who are stopped multiple times have their race inconsistently perceived across stops, for example, Hispanic in one stop and white in another. [Following previous literature on police discrimination, we use “race” throughout to refer to race/ethnicity (*3*, *4*, *43*, *44*); Hispanic identity is often considered an ethnicity and not a race (*45*)]. We focus our analysis on drivers perceived as both white and Hispanic because these drivers constitute 75% of drivers whose race is inconsistently perceived. We find that these drivers are more likely to be searched or arrested by police when they are perceived as Hispanic than when they are perceived as white, suggesting bias against Hispanic drivers. These disparities are statistically and practically significant: for example, the disparity in search rates is 24% of the overall search rate.

A key advantage of our method, relative to past benchmarking tests for bias, is that it does not require us to control for all confounds that might influence search behavior for legitimate reasons, which are myriad and potentially unobserved. Rather, our test requires us only to control for the much more limited set of confounds that influence search behavior, influence perceived race, vary for the same person over time, and do not suggest bias. For example, the driver’s perceived skin tone plausibly satisfies the first three criteria, but not the fourth (*46*–*48*). We hence show that our core finding is robust to controlling for the variables that satisfy all four of these criteria: for example, location, time, and individual officer effects.

As a concrete example of a variable that is necessary to control for under a traditional benchmarking approach, but not under our test, consider “the driver is behaving in a threatening manner.” This variable could influence the search decision for legitimate reasons, and it is thus important to control for under a traditional benchmarking approach; however, it is often impossible to control for this variable because it is unrecorded in the dataset, a major weakness of traditional benchmarking approaches that often precludes their use as reliable tests for bias (*3*, *4*). In contrast, it is not necessary to control for this variable when using our approach, because for it to explain the disparities observed under our test, officers would have to associate threatening driver behavior not only with the need to search them but also with whether the driver is Hispanic—and if the officer assumes that people who are behaving in a threatening way are more likely to be Hispanic, that is itself an indicator of officer bias. More broadly, our approach removes the need to control for search-relevant external variables (such as threatening behavior) if the officer’s use of these variables in classifying driver race would itself indicate bias by foreseeably stigmatizing or harming the classified group. In general, our test does not require us to control for variables that would (i) suggest bias if they were used to infer someone’s race (such as threatening behavior) or (ii) suggest bias if they were used to inform the search decision (such as skin color).

Beyond policing, our method can be applied to test for bias in the many other settings— including health care, survey, criminal justice, and child welfare datasets (*32*, *34*–*37*, *49*–*51*)—where race, gender, or other measures of identity are perceived rather than self-reported and the same person is observed multiple times. We conclude by discussing several specific further settings where our method can be applied.

## RESULTS

Our test quantifies whether the same person is treated differently when their race is perceived differently. We first describe the motivation for this test and then provide a mathematical description of how we implement it.

### Motivation

We motivate our test with a conceptual model similar to the constructivist model of discrimination proposed in (*18*). (In our empirical analysis, we do not actually attempt to estimate the components of this conceptual model; rather, we use it to provide theoretical justification for our analysis.) For concreteness, we describe the model in the context of our specific empirical setting—assessing whether the same person is more likely to be searched by police when they are perceived as Hispanic than when they are perceived as white—but our approach naturally extends to other decision-making settings.

We assume that when an officer stops a driver, they observe features ***X***—the appearance and behavior of the driver, type of car, time and date of the stop, and so on—and, on the basis of those features, perceive the driver’s race, r(X)∈[0,1] , on a binary continuum that ranges from white [ r(X)=0 ] to Hispanic [ r(X)=1 ]. [For simplicity, we follow (*18*) in modeling race as a binary continuum, but our approach is also applicable to more than two racial categories.] After perceiving the driver’s race, as in past models of police searches (*3*, *4*, *44*), the officer infers the probability that the driver is carrying illegal contraband—c(X, r)∈[0,1]—and searches the driver if this probability exceeds a threshold t(X, r)∈[0,1] . Both the inferred probability *c* and threshold *t* may vary both by *r* and by *X*.

If we find that the same person is more likely to be searched by police when they are perceived as Hispanic than when they are perceived as white, three possibilities might explain this result. Under the first two possibilities, the person’s perceived race directly affects officer behavior; under the third possibility, some third variable influences both perceived race and officer behavior.

1) Police infer that the same person is more likely to be carrying contraband when they perceive them as Hispanic. In other words, c(X,r=1)>c(X,r=0) . This constitutes statistical racial discrimination (*52*), which is generally illegal (*53*), including in the police search context that we study (*4*).

2) Police apply a lower threshold for searching the same person when they perceive them as Hispanic. In other words, t(X,r=1)<t(X,r=0) . This constitutes taste-based racial discrimination (*52*), which is also illegal both in general and in the police search context that we study (*4*).

3) Some aspect of *X* changes for the same person from stop to stop and affects both their perceived race and the officer’s search behavior. This possibility requires further investigation, because there are a limited number of possibilities for this time-varying *X* that might not suggest bias on the part of decision-makers. For example, if the same driver interacts with both Hispanic and white police officers and if Hispanic officers are both likelier to perceive someone as Hispanic and likelier to conduct searches, then drivers would be more likely to be searched when perceived as Hispanic even in the absence of bias by any individual officer; the race of the decision-maker thus acts as the time-varying component of *X*. Note that a major benefit of our method, in contrast to past benchmarking tests for bias, is that it does not require us to control for all *X* that might influence search behavior for legitimate reasons (*4*). Rather, our test requires us only to control for the much more limited set of *X* satisfying four conditions: they (i) influence search behavior, (ii) influence perceived race, (iii) vary for the same person over time, and (iv) do not suggest bias. For example, the driver’s perceived skin color plausibly satisfies the first three criteria, but not the fourth (because perceived skin color should not be a factor in the search decision). Similarly, whether the driver behaves in a threatening manner would not satisfy the fourth criterion because it should not affect the officer’s perception of their race. More generally, as discussed above, we do not need to control for variables that would indicate bias if they were used to infer someone’s race or to decide whether to search them. In our sensitivity analyses below, we explicitly check for these nonbias explanations.

Our conceptual model follows past work in assuming that the officer perceives the driver’s race before making the search decision, but it is also possible that these judgments occur in the opposite order, i.e., that the officer first decides to search and then perceives the driver’s race. We further discuss this possibility, and why it would also indicate a problematic bias, after presenting our empirical results.

### Empirical implementation

As above, we introduce notation in the context of our specific empirical setting, but our approach naturally extends to other decision-making settings and to more than two race groups. Let *i* index people and *t* index time. Let $y_{\mathrm{it}}\in \left\{0,1\right\}$ be a binary variable indicating whether the person was searched by police, let $r_{\mathrm{it}}\in \left\{0,1\right\}$ be a binary variable denoting whether their race was perceived as Hispanic ( $r_{\mathrm{it}}=1$ ) or white ( $r_{\mathrm{it}}=0$ ), and let $X_{\mathrm{it}}$ denote covariates that may vary by person and over time. We estimate the following model

$$y_{\mathrm{it}}=\alpha_{i}+\beta X_{\mathrm{it}}+\delta r_{\mathrm{it}}+\epsilon_{\mathrm{it}}$$

The observed data are $\left\{y_{\mathrm{it}},X_{\mathrm{it}},r_{\mathrm{it}}\right\}$ ; the parameters to be estimated are the person fixed effects $\alpha_{i}$ , the coefficients on controls β , and the main parameter of interest δ , which quantifies how the same person’s likelihood of being searched differs when the perception of their race differs.

In our primary specification, we estimate coefficients using a linear fixed effects model and report SEs clustered at the driver level. Because our empirical setting analyzes a binary outcome variable $y_{\mathrm{it}}$ (whether the police conduct a search), this corresponds to a linear probability model. These models are often used in fixed effects settings with binary variables (*54*, *55*) due to their simplicity and interpretability. However, to confirm that our results are robust to misspecification caused by the binary outcome, we additionally fit several models specifically designed for binary outcomes (figs. S2 and S3). Our results remain similar with, and our framework naturally accommodates, these alternate specifications.

### Policing data

We use data and data processing code from the Open Policing Project (*3*), a study of police traffic stops across the United States. We analyze state patrol traffic stop data from the three states—Arizona, Colorado, and Texas—which provide the information required to track the same driver across multiple stops, as our approach requires, and filter for drivers stopped multiple times. For example, in Colorado, we link a driver across multiple stops using name and date-of-birth (see Materials and Methods for further details). A total of 9% of drivers stopped multiple times have their race inconsistently perceived across stops (for example, Hispanic in one stop and white in another). The large majority of these inconsistently perceived drivers (75%) are recorded as both Hispanic and white (as opposed to some other combination of race groups); we thus filter for these drivers, who comprise our final analysis sample of 54,170 drivers and 157,755 stops. The same driver is likelier to be classified as Hispanic when the stop occurs in a county with a larger Hispanic population, consistent with past work that finds that perceptions of someone’s race correlate with the demographics of their local area (*56*); we also observe smaller, although statistically significant, time effects (Supplementary Materials; fig. S4).

We use the search rate—i.e., a driver’s probability of being searched after being stopped by the police—as our metric of negative treatment, following past work (*3*, *4*, *9*, *44*, *57*). (We confirm that results remain similar when using the arrest rate—i.e., the fraction of stops which result in arrest—as an alternate metric.) In all three states, the search rate is higher for our sample of inconsistently perceived drivers when they are perceived as Hispanic than when they are perceived as white (Arizona: 4.2% versus 3.2%; Colorado: 0.5% versus 0.4%; Texas: 1.8% versus 1.5%). Table 1 provides descriptive statistics for the sample, and Materials and Methods fully describes all data processing procedures.

**Table 1.** Descriptive statistics of the dataset. Each section of the table reports statistics for increasingly nested subsets of the data: all drivers with multiple stops, drivers with multiple stops and inconsistently perceived race, and inconsistently perceived drivers recorded specifically as white and Hispanic.

	Arizona	Colorado	Texas	Overall
All drivers with multiple stops				
Drivers	275,412	345,774	184,257	805,443
Stops	648,797	927,533	430,843	2,007,173
Drivers with multiple stops and inconsistently perceived race				
Drivers	30,065	32,565	9473	72,103
% of all multiply stopped drivers	10.9%	9.4%	5.1%	9.0%
Stops	81,625	100,760	23,480	205,865
% of all multiply stopped driver stops	12.6%	10.9%	5.4%	10.3%
Drivers perceived as both white and Hispanic				
Drivers	19,285	27,423	7462	54,170
% of all inconsistently–perceived drivers	64.1%	84.2%	78.8%	75.1%
Stops	52,771	86,177	18,807	157,755
% of all inconsistently perceived driver stops	64.7%	85.5%	80.1%	76.6%
Search rate when perceived as Hispanic	4.2%	0.5%	1.8%	1.9%
Search rate when perceived as white	3.2%	0.4%	1.5%	1.5%

### Estimates of bias

**Fig. 1.**
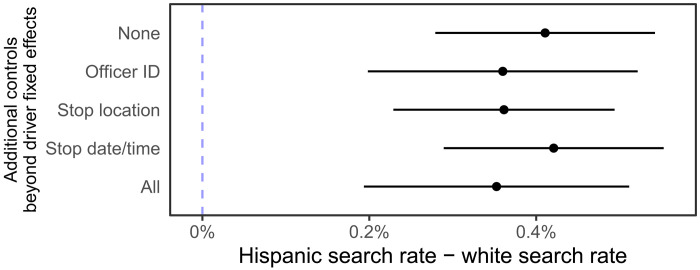
Estimated increase in the search rate, in percentage points, when the same driver is perceived as Hispanic as opposed to white using a linear probability model. All estimates include driver fixed effects. The 95% confidence intervals are plotted with SEs clustered at the driver level. Estimates remain similar when including controls for officer identity, stop location, and stop date and time.

Figure 1 presents results from our primary specification, a linear probability model, pooling data across all three states and clustering SEs at the driver level (table S1 reports full regression coefficients). With driver fixed effects, but no additional controls, we estimate that the same driver is 0.4 percentage points (pp) more likely to be searched when they are perceived as Hispanic than when they are white, suggesting bias against Hispanic drivers [95% confidence interval (CI), 0.3 to 0.5 pp; $P<10^{-4}$ ). This is not only a statistically significant but also a practically significant effect: It is 24% of the average search rate across all states (1.7%).

We conduct several sensitivity analyses. (We describe three in detail here; the Supplementary Materials reports additional sensitivity analyses, including removing each state from the analysis, and filtering for drivers with varying numbers of stops.) First, results remain robust to inclusion of additional controls. We assess the sensitivity of the results when including the battery of controls that are available across all three states and used in previous analyses of this dataset (*58*): stop location (encoded as county) and stop date and time (encoded as stop year, stop quarter, weekday, and stop hour binned into eight 3-hour bins). Stop location can influence racial perception (because driver demographics correlate with location) as we show in fig. S4, and as past work has also found (*35*); stop location could also influence search behavior (*3*, *4*, *43*, *44*, *58*). Similarly, the time of day can influence both racial perception [if, for example, lighting conditions differ (*3*, *12*); fig. S4] and search behavior. Estimates remain similar (Fig. 1) when including these additional controls. We also assess whether our results remain robust when including a fixed effect for the officer conducting the stop, since officer identity might affect both search behavior and perception of the driver’s racial identity; our estimates remain similar (Fig. 1). A final explanation for our results that would not necessarily suggest bias is that searches result in longer stops, giving the officer more time to perceive the race of the driver and altering their perception of the driver’s race. We investigate this explanation using data from Arizona, the only state that records stop duration. The discrepancy in search rates remains large when we include controls for stop duration (0.6 pp, 95% CI, 0.3 to 0.9 pp, $P<10^{-4}$ ), suggesting that this does not explain the results.

Second, we examine the robustness of our results to using an alternate definition of negative treatment—specifically, whether the driver was arrested—in the states for which arrest data are available, Arizona and Colorado. We find similar evidence of bias in arrest rates (the fraction of stops which result in arrests; fig. S1): We estimate that the same driver is 0.4 pp (95% CI, 0.2 to 0.6 pp, $P<10^{-4}$ ) more likely to be arrested when they are perceived as Hispanic than when they are white.

Third, we show that our results remain robust when using alternate statistical models designed for binary outcomes: Specifically, a fixed effects generalized linear model with a logit link (fig. S3) and a conditional logistic regression (fig. S3). Collectively, these sensitivity analyses show that our finding of bias against Hispanic drivers remains robust across alternate sets of controls, outcome definitions, and statistical models.

## DISCUSSION

We propose a test for bias that quantifies whether the same person is treated differently when their race is perceived differently. We apply our method to assess bias in searches after state patrol stops across three states, finding that the same driver is more likely to be searched or arrested when they are perceived as Hispanic than when they are perceived as white.

Several points are important to keep in mind when interpreting our results. First, our test analyzes a subset of individuals—those whose race is differently perceived across multiple encounters. This population is one of substantial interest given past research on multiracial and racially ambiguous individuals (*19*, *30*, *33*, *59*–*62*), rendering these results important in their own right. However, our findings should not be assumed to generalize to all white and Hispanic drivers: For example, our test might fail to show evidence of bias even if Hispanic drivers as a whole face bias. Further, if the population we analyze is highly nonrepresentative (e.g., in terms of stop reasons or stop outcomes), our findings might not generalize to other populations. To assess this, we compare our analyzed population to two relevant comparison populations—(i) all white and Hispanic drivers and (ii) all white and Hispanic drivers stopped multiple times—in terms of both stop circumstances and stop outcomes (Supplementary Materials). These analyses suggest qualitatively similar trends across all three populations—particularly, the most common stop reasons are identical across all three populations, and drivers perceived as Hispanic are searched and arrested at higher rates than drivers perceived as white, suggesting that this trend holds robustly and is not merely due to a nonrepresentative population. Future analyses of bias on this dataset should assess the extent to which our findings generalize. Tests for bias often derive their estimates from different subsets of individuals—for example, the “veil of darkness” test analyzes individuals stopped near sunset (*12*), and outcome tests (*4*, *63*) typically analyze only individuals searched by the police—and thus have different limitations. Hence, the most robust analyses will combine multiple complementary tests for bias, making our test a useful addition to other tests for bias that analyze different subsets of the population.

Second, we apply our test to analyze drivers perceived as both white and Hispanic; future work should assess the extent to which our approach generalizes to other pairs of race groups. While past literature attests to the unique dynamics underlying Hispanic identification (*64*), it also shows that non-Hispanic groups can be inconsistently perceived as well (*34*, *39*, *65*, *66*): For example, Vargas and Stainback (*66*) find that 8% of Asian Americans and 7% of Black Americans (as compared to 13% of Hispanic Americans) report being perceived as a race group that differs from their own self-identified race. Past literature and our own data suggest that, in most circumstances, only a minority of individuals will be inconsistently perceived and that the groups for which this occurs will vary depending on local context and demographics: For example, we study three states with large Hispanic populations, and this likely contributes to the fact that we are best-powered to study drivers perceived as both Hispanic and white. In other contexts, however, other pairs of race groups may be inconsistently perceived.

Third, while under our model the officer first perceives an individual’s race and then decides whether to search them, these two judgments could also occur in the opposite order (*29*). Racial judgments occur extremely quickly (*28*, *67*), perhaps making the reverse order (in which the search decision precedes the racial judgment) less likely; however, it is possible that events that occur throughout the stop, including the behavior of the driver, influence the officer’s racial perception. This reverse order would add nuance to the interpretation of our results because it would suggest that the officer’s decision to search or arrest the driver caused them to perceive the driver as Hispanic, rather than the other way around. This would also be a concerning bias—namely, that the officer’s perception of Hispanic people includes “people I search and arrest,” which likely leads them to engage in statistical discrimination when deciding whether to search or arrest. We also note that similar ambiguities are common in tests for bias: For example, outcome tests can show bias either because officers misestimate the probability that drivers carry contraband or correctly estimate the probability and apply different thresholds when searching drivers of different races.

A fourth important point to bear in mind when applying our method is that it requires data on perceived race as opposed to self-identified race. We hence verify, by reaching out to the state patrols of all three states whose data we analyze, that race data are recorded according to the perception of the officer. In other settings, we similarly recommend reaching out to domain experts familiar with the data recording process before applying our method.

Fifth, our analysis considers the population of drivers stopped by police, and the stop decision may itself be the product of bias (*3*). Previous work has highlighted the complexities that arise when estimating bias in these selected populations (*68*) and raised concerns about whether it is possible to estimate relevant measures of bias; however, subsequent work has clarified that estimands of interest remain well-identified under appropriate assumptions (*69*), and analyses of bias among the stopped population continue to be widely conducted (*3*, *70*, *71*). Nonetheless, bias in police searches, while important, is only one of the ways that bias can manifest throughout a multistage policing pipeline.

Last, past work shows that police may sometimes strategically misclassify minorities as white to conceal disparate rates of negative treatment (*3*, *72*, *73*): In other words, reported race might not represent perceived race, but rather the officer’s deliberate misclassification. This misclassification would lead us to underestimate the extent of bias against Hispanic drivers (since officers will be more likely to strategically misclassify them as white when searching them). Hence, even in the presence of strategic misclassification, our finding of bias against Hispanic drivers likely holds. (We also minimize the impact of possible misclassification by filtering out time periods where past work suggests it occurred; see Materials and Methods for full details.)

While we illustrate our approach by assessing racial bias in policing, our core contribution is a method that is much more broadly applicable to testing for bias in datasets that (i) track the same individual over time; (ii) record perceptions of the person’s race or other sensitive attributes, such as gender, along with sufficiently rich data on controls to rule out nonbias explanations; and (iii) are large enough to analyze the subset of individuals with inconsistently perceived identity. These datasets occur in many other settings (*32*, *34*–*37*, *42*, *49*–*51*); we conclude by discussing four in more detail. First, past work documents how child welfare datasets meet the three aforementioned criteria: They track repeated interactions with the same child over time; often record the child’s race as perceived by investigators, along with rich contextual information; and are of comparable size to the data we analyze here (*34*). Second, criminal justice datasets (e.g., court and prison records) track repeated interactions with the same individual over time; often record race as perceived by justice agency personnel, along with detailed controls; and are comparable in size to the data we analyze here (*35*). Third, some labor market datasets track wage and employment histories for the same person over time; record race as perceived by the employer, along with rich controls; and are large enough to support analogous analyses (*42*). Fourth, health care datasets (e.g., from electronic health records) contain multiple records from the same patient over time; often record race as perceived by health care workers (*50*, *74*–*77*), along with detailed information about the patient’s health state; and may span millions or tens of millions of patients (*36*, *78*). Quantifying bias in all of these settings is of substantial interest (*34*, *35*, *42*, *78*–*81*). These settings illustrate the breadth of datasets to which our method applies, providing a rich set of directions for future work.

## MATERIALS AND METHODS

### Data processing

Our analysis relies on data and data processing procedures from the Open Policing Project (*3*). We analyze state patrol stops from Arizona (2010–2015), Colorado (2010–2016), and Texas (2016), which are the three states that provide the requisite data to match the same driver across multiple stops. Analysis was deemed exempt from review by the Cornell Institutional Review Board (IRB) (IRB0148948).

### Confirming race data represents officer perception

Our method requires data on perceived race as opposed to self-identified race. We hence verified, by reaching out to the relevant departments of all three states whose data we analyzed, that race data are recorded according to the perception of the officer. The Arizona Department of Public Safety confirmed that the data represented officer perception: “we rely on the trooper’s perception, utilizing their best judgement under the circumstances.” The Colorado State Patrol told us that “[t]he race and ethnicity data is in fact the trooper’s perception.” The Texas Highway Patrol provided us with an excerpt from their policy manual, which instructs officers to “[u]se your best judgement in determining the race or ethnicity of the individual.” We note that the situation in Texas is somewhat more ambiguous because in November 2015, several news articles (*73*, *82*) suggested that Texas officers were instructed to ask for a driver’s race. However, the response we received from the Texas Highway Patrol, combined with the fact that driver race was fairly frequently inconsistently recorded in our data even over a short time period, suggests that this was not consistently done and that the race was recorded based on officer perception. We confirm that our results are robust to excluding data from Texas (fig. S5). Overall, our investigations confirm that the recorded data represent officer perception in all three states.

### Variation in estimates across states

We assess how our results vary when dropping each state from the analysis dataset (fig. S5). Our primary estimate (with driver fixed effects, but no other controls) when analyzing all three states is that the white-Hispanic disparity in search rate is 24% of the overall search rate. When excluding Texas, this disparity is 25% ( $P<10^{-4}$ ); excluding Arizona, it is 15% ( P=0.07 ); and excluding Colorado, it is 26% ( $P<10^{-4}$ ). The confidence intervals become considerably wider (and the point estimates somewhat smaller) when dropping Arizona, due both to the reduction of sample size and the lower search rate in the other two states; in our primary specification, the effect when dropping Arizona is significant at only the *P* = 0.1 level, and in some specifications, the estimate when dropping Arizona is not statistically significant. However, in all cases, the estimates are directionally consistent, and the CIs when dropping Arizona overlap the original point estimates.

### Removing stops with deliberately misclassified driver race

In late 2015, investigative journalists provided evidence that Texas police were deliberately misclassifying Hispanic drivers as white (*72*, *73*), something the agency subsequently corrected. Consequently, we remove Texas data recorded before 2016, since the evidence suggests that data contain instances of deliberate misrecording by the officer, as opposed to the officer’s true perception. From 2016 onward, the data recording by the police appear much more reliable: Specifically, the fraction of drivers with Hispanic surnames who are recorded as white falls sharply in December 2015, just after the investigative journalists published their report. This gives us confidence that data from 2016 onward are more reliably recorded and, particularly, that inconsistencies in the recording of driver race result from good-faith misperception as opposed to deliberate misrecording. We do not know of evidence that driver race was deliberately misclassified in Arizona or Colorado, the other two states we analyze (*58*). It is possible that there is residual deliberate misreporting we are unable to identify. However, any such misreporting would likely lead us to underestimate the extent of bias against Hispanic drivers (since officers would be more likely to deliberately misclassify them as white when searching them to conceal racial bias). Hence, even in the presence of deliberate misclassification, our finding of bias against Hispanic drivers likely remains robust.

### Factors that correlate with whether the same driver is perceived as white or Hispanic

We investigate the factors that correlate with whether the same driver is perceived as white or Hispanic using a fixed effects regression directly analogous to that used to generate our primary results. Specifically, following the notation in the main text, we estimate the following fixed effects model

$$r_{\mathrm{it}}=\alpha_{i}+\beta X_{\mathrm{it}}+\epsilon_{\mathrm{it}}$$

where the dependent variable, $r_{\mathrm{it}}\in \left\{0,1\right\}$ , is whether the driver’s race is perceived as Hispanic $\left(r_{\mathrm{it}}=1\right)$ or white ( $r_{\mathrm{it}}=0$ ). The parameters to be estimated are the driver fixed effects $\alpha_{i}$ and the coefficients on controls β . We include controls for stop date and time (encoded as stop year, stop quarter, weekday, and stop hour binned into eight 3-hour bins, as in our main specification), and the Hispanic proportion of the county where the stop occurs (as estimated from 2011 to 2015 5-year American Community Survey data, the most relevant time period for our analysis).

Figure S4 plots the estimated regression coefficients. The largest effect is that the same driver is significantly more likely to be perceived as Hispanic when the stop occurs in a county with a larger Hispanic proportion, with a one SD increase in the county Hispanic proportion corresponding to a 12 pp increase in the probability that the same driver is perceived as Hispanic ( P<0.001 ). Similar effects have been observed in prior work (*56*). (Equivalently, a 10 pp increase in the county Hispanic proportion corresponds to a 6 pp increase in the probability that the driver is perceived as Hispanic).

The time trends, although sometimes statistically significant, generally have smaller magnitudes. We do observe a 5 to 10% drop in the probability a driver is classified as Hispanic in later years compared to 2010. To confirm that this does not affect our results, we rerun our analysis dropping data from 2010. Results remain very similar: Our estimated white-Hispanic difference in the search rate (with driver fixed effects, but no other controls) when dropping 2010 is 0.45 (95% CI, 0.31 to 0.60); when including 2010, the estimate is 0.41 (95% CI, 0.28 to 0.54). Results for the other regression specifications are similarly stable. Estimates remain stable both because stops from 2010 comprise only 10% of the dataset and because the change in the fraction classified as Hispanic is not large relative to the overall mean.

### Assessing representativeness of analyzed population

Our estimates of bias come from analyzing drivers who are stopped multiple times and who are perceived as both white and Hispanic. To assess the representativeness of our analyzed population, we compare it to two other relevant populations: (i) all Hispanic or white drivers and (ii) Hispanic or white drivers stopped multiple times. We compare both reasons for the stop and outcomes of the stop (as measured by search and arrest rates).

#### Analysis of stop reasons

Because each state records stop reasons in different formats, we analyze stop reasons for each state separately. In all three states, the top three stop reasons are the same for both our analyzed population and the two comparison populations, suggesting similar stop circumstances. In Arizona, the top three stop reasons are “Speeding,” “Other reasons,” and “Moving violations”; in Colorado, “Speeding,” “Safe movement,” and “Lights”; and in Texas, “Speeding Over Limit (#),” “Speeding-10% or More Above Posted Speed (#),” and “Operate Motor Vehicle Without License Plates (or With One Plate).”

#### Analysis of stop outcomes

We next compare search rates and arrest rates in our analyzed population to those in the two comparison populations. (We omit Texas from the arrest rate analysis because it does not provide arrest data.) In all three populations, drivers perceived as Hispanic are searched at higher rates than drivers perceived as white, suggesting that this trend holds robustly and is not merely due to a nonrepresentative population. The search rate in our analyzed population of inconsistently perceived drivers is 1.5% (95% CI, 1.4 to 1.6%) for drivers perceived as white compared to 1.9% (95% CI, 1.8 to 2.0%) for drivers perceived as Hispanic; the search rate in multiply stopped drivers is is 1.2% (95% CI, 1.1 to 1.2%) for drivers perceived as white compared to 2.7% (95% CI, 2.6 to 2.8%) for drivers perceived as Hispanic; and the search rate in all drivers is 1.5% (95% CI, 1.5 to 1.5%) for drivers perceived as white compared to 3.6% (95% CI, 3.6 to 3.6%) for drivers perceived as Hispanic. Similarly, the arrest rate in our analyzed population of inconsistently perceived drivers is 2.1% (95% CI, 1.9 to 2.2%) for drivers perceived as white compared to 2.4% (95% CI, 2.3 to 2.6%) for drivers perceived as Hispanic, the arrest rate in multiply stopped drivers is 1.7% (95% CI, 1.7 to 1.7%) for drivers perceived as white compared to 2.4% (95% CI, 2.4 to 2.5%) for drivers perceived as Hispanic, and the arrest rate in all drivers is 1.7% (95% CI 1.7%–1.7%) for drivers perceived as white compared to 3.0% (95% CI, 2.9 to 3.0%) for drivers perceived as Hispanic. The white-Hispanic search and arrest rate gaps are somewhat larger in the two comparison populations relative to our analyzed population; this is an expected consequence of the fact that our analyzed population contains a single group of drivers perceived as both white and Hispanic, as opposed to distinct groups of drivers perceived as white and Hispanic. The differences between these distinct groups may also include confounds; a primary benefit of our method is that it reduces this concern. Overall, the fact that search and arrest rate disparities hold robustly across both comparison populations suggests that our finding of bias is not merely the product of analyzing a nonrepresentative population.

### Matching drivers across multiple stops

The data available to match the same driver across multiple stops vary by state. In Arizona, we use the first and last name of the driver and their vehicle style and year, which are available for 99% of stops; in Colorado, the driver’s first and last name and date of birth, which are available for 85% of stops; and in Texas, the driver’s first and last name and complete address, which are available for 96% of stops.

Our analysis requires the fields we use to correctly differentiate distinct drivers (i.e., two different people should very rarely have the same values). In Texas, this is very likely to be true: Two different people are very unlikely to have the same full name and full address. In Colorado, past analyses of the probability two people share the same full name and birth date (*83*) also implies that this is very likely to be true in a sample of our size.

In Arizona, we are not aware of past work analyzing the probability two distinct people share the same first name, last name, vehicle style, and vehicle year. However, the descriptive statistics of our dataset suggest that these fields are indeed sufficient to differentiate distinct drivers. In particular, if distinct drivers were frequently being incorrectly combined in Arizona, then we would expect its fraction of drivers with multiple stops to be higher, and its number of stops for each driver to be higher, than in Colorado, where we have access to fields that reliably differentiate distinct drivers and whose data spans a similar time period. We observe the opposite: Only 15% of drivers are stopped multiple times in Arizona, as opposed to 22% in Colorado, and the number of stops per driver in Arizona is 1.2, as opposed to 1.4 in Colorado. This suggests that incorrectly matched drivers are not skewing Arizona data. As a final precaution against incorrectly matched drivers, we remove the small proportion (0.1%) of drivers who are recorded as having more than 10 stops to prevent drivers who have been incorrectly matched many times from skewing the results. This does not affect our conclusions; similarly, filtering for drivers with exactly two stops yields similar estimates of bias (fig. S6).

## Supplementary Materials

### This PDF file includes:

Table S1 Figs. S1 to S6 References
