# Supplementary material for: Testing for racial bias using inconsistent perceptions of race
Source: Sci Adv. 2025 Nov 19;11(47):eadx5829. doi: 10.1126/sciadv.adx5829 (PMC13142046; doi:10.1126/sciadv.adx5829)
Supplement: Supplementary file 1 — Table S1 Figs. S1 to S6 References [file sciadv.adx5829_sm.pdf]

Science Advances
AAAS

Supplementary Materials for

Testing for racial bias using inconsistent perceptions of race

Nora Gera and Emma Pierson

Corresponding author: Emma Pierson, emmapierson@berkeley.edu

Sci. Adv. **11**, eadx5829 (2025)
DOI: 10.1126/sciadv.adx5829

This PDF file includes:

Table S1
Figs. S1 to S6
References

Table S1: **Full regression results for the models depicted in Figure 1.** Coefficients report the estimated effect on the search rate in percentage points.

Controls beyond driver fixed effects	*None*	*Officer*	*Location*	*Date/Time*	*All*
Hispanic	0.411*** (0.067)	0.360*** (0.083)	0.361*** (0.068)	0.421*** (0.067)	0.352*** (0.081)
Year 2011				0.066 (0.122)	0.134 (0.120)
Year 2012				-0.013 (0.124)	0.054 (0.127)
Year 2013				-0.091 (0.123)	0.014 (0.131)
Year 2014				-0.435*** (0.126)	-0.432** (0.134)
Year 2015				-0.181 (0.138)	-0.262· (0.146)
Year 2016				-0.482* (0.188)	-0.476* (0.201)
Q2				-0.223* (0.109)	-0.222* (0.105)
Q3				-0.275* (0.113)	-0.228* (0.111)
Q4				-0.023 (0.113)	-0.016 (0.111)
Monday				-0.064 (0.140)	-0.155 (0.141)
Tuesday				-0.105 (0.136)	-0.138 (0.137)
Wednesday				-0.089 (0.138)	-0.093 (0.138)
Thursday				-0.172 (0.136)	-0.220 (0.134)
Saturday				0.172 (0.141)	0.106 (0.137)
Sunday				0.158 (0.144)	0.112 (0.143)
3 AM - 6 AM				-0.347 (0.265)	0.004 (0.249)
6 AM - 9 AM				-1.29*** (0.206)	-0.612** (0.191)
9 AM - 12 PM				-1.31*** (0.213)	-0.760*** (0.200)
12 PM - 3 PM				-1.08*** (0.203)	-0.514** (0.190)
3 PM - 6 PM				-1.14*** (0.208)	-0.543** (0.190)
6 PM - 9 PM				-0.988*** (0.204)	-0.517** (0.189)
9 PM - 12 AM				-0.711*** (0.206)	-0.428* (0.190)
Fixed effects					
Driver	Yes	Yes	Yes	Yes	Yes
Officer		Yes			Yes
County			Yes		Yes

Standard errors clustered at the driver level in parentheses

*Signif. Codes: ***: 0.001, **: 0.01, *: 0.05, .: 0.1*

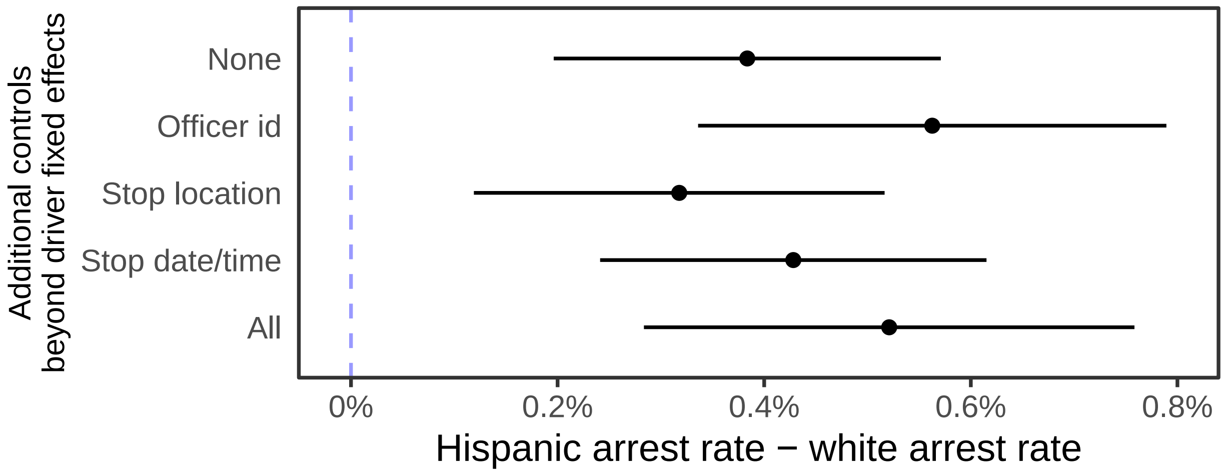

Figure S1: **Estimated increase in the *arrest* rate in percentage points (as opposed to the search rate, as in our primary specification) when the same driver is perceived as Hispanic as opposed to white, using a linear probability model.** The arrest rate is defined as the fraction of stops which result in arrests. All estimates include driver fixed effects. 95% confidence intervals are plotted with standard errors clustered at the driver level. Estimates use Colorado and Arizona data because Texas does not provide arrest data. The finding of bias against Hispanic drivers remains robust when using this alternate outcome, and including controls for officer, stop location, and stop date/time.

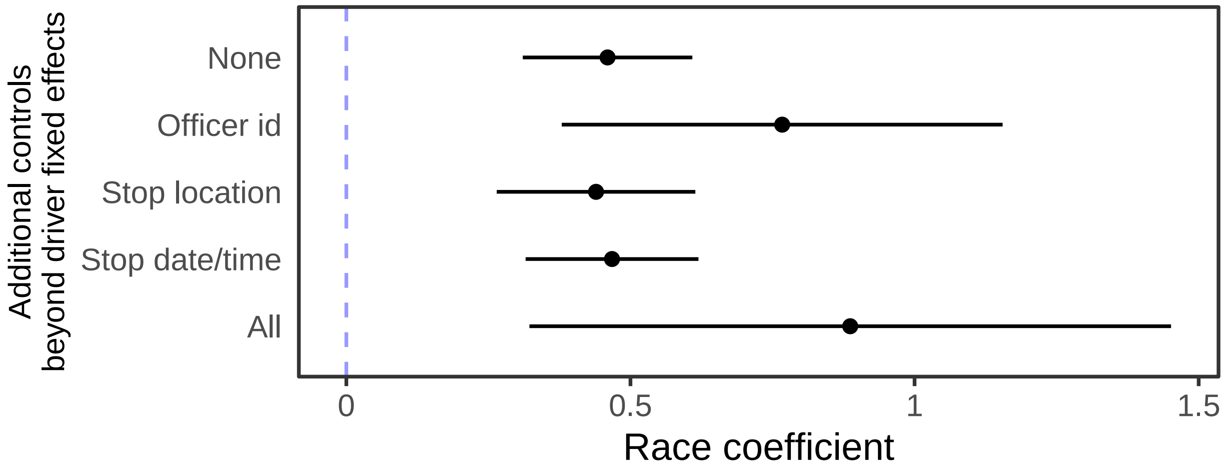

Figure S2: **Estimates from a fixed effects generalized linear model with a logit link.** The horizontal axis plots the coefficient on driver race = Hispanic after controlling for driver fixed effects. The finding of bias against Hispanic drivers remains robust when using this alternate statistical model, and including controls for officer, stop location, and stop date/time.

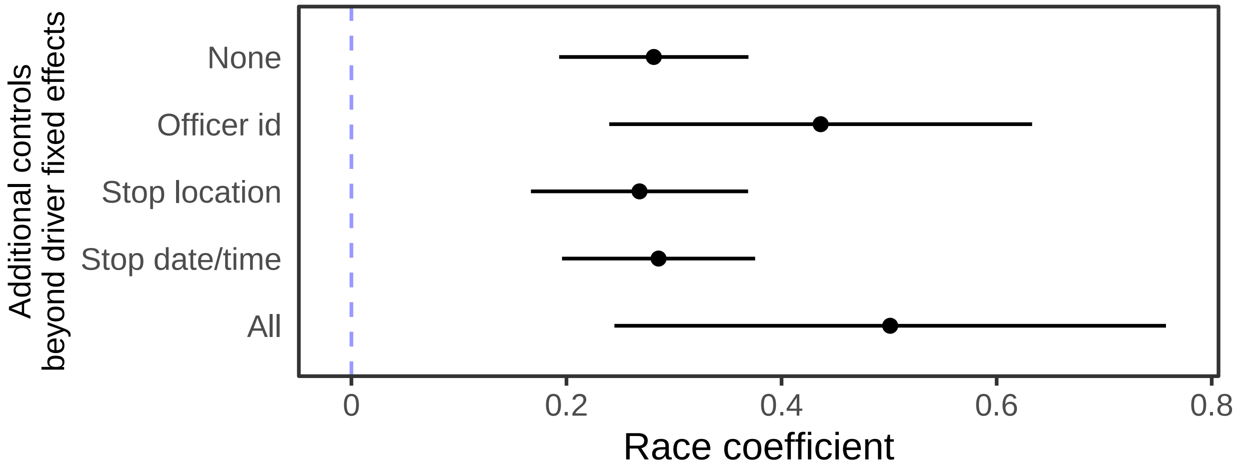

Figure S3: **Estimates from a conditional logistic regression model (*84*) with a stratum for each driver.** The horizontal axis plots the coefficient on driver race = Hispanic. The finding of bias against Hispanic drivers remains robust when using this alternate statistical model, and including controls for officer, stop location, and stop date/time.

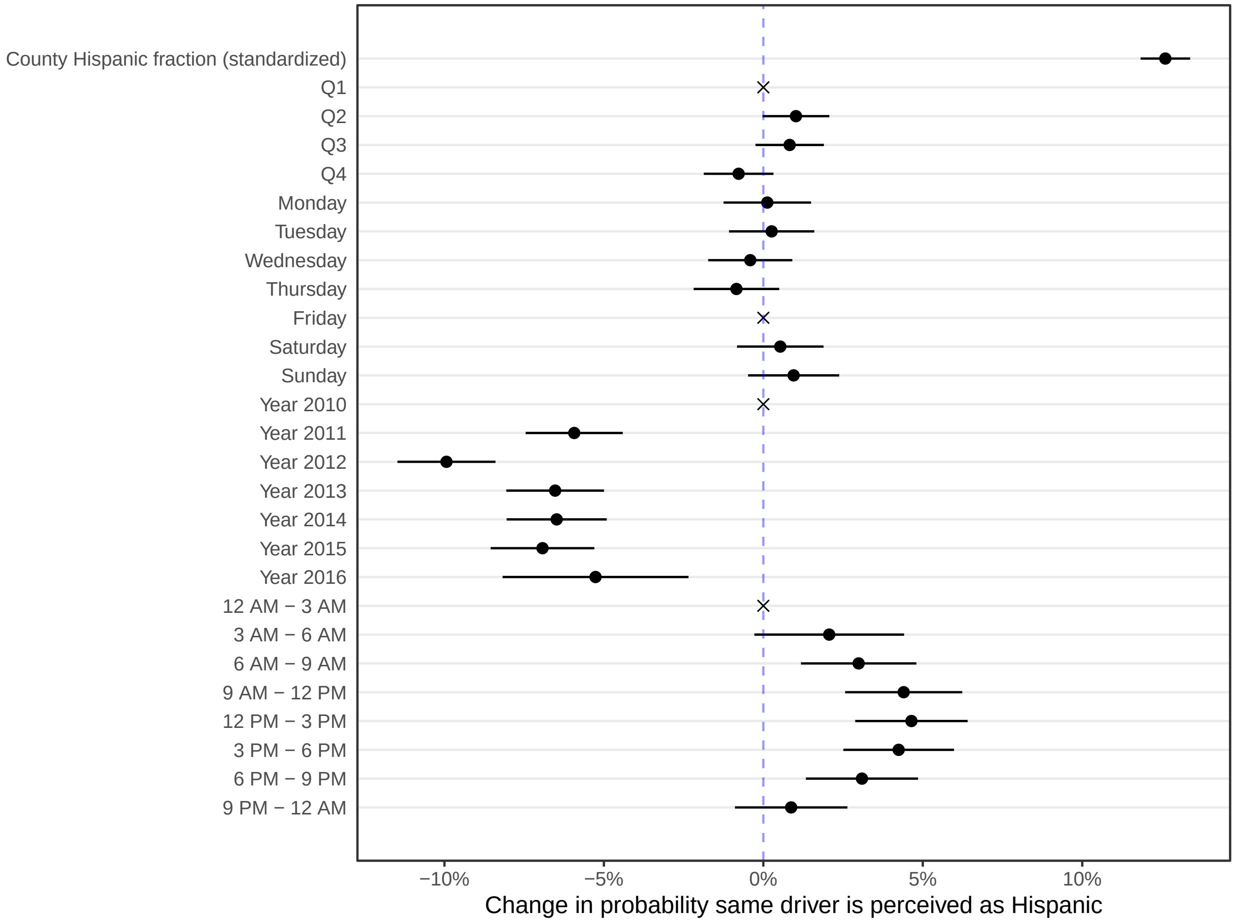

Figure S4: **Estimated change in the probability that the same driver is classified as Hispanic, in percentage points, using a linear probability model.** All estimates include driver fixed effects. 95% confidence intervals are plotted with standard errors clustered at the driver level. County Hispanic fraction is reported in units of standard deviations across the counties in Colorado, Arizona, and Texas; all other covariates are binary indicators, with reference (omitted) levels denoted by x's.

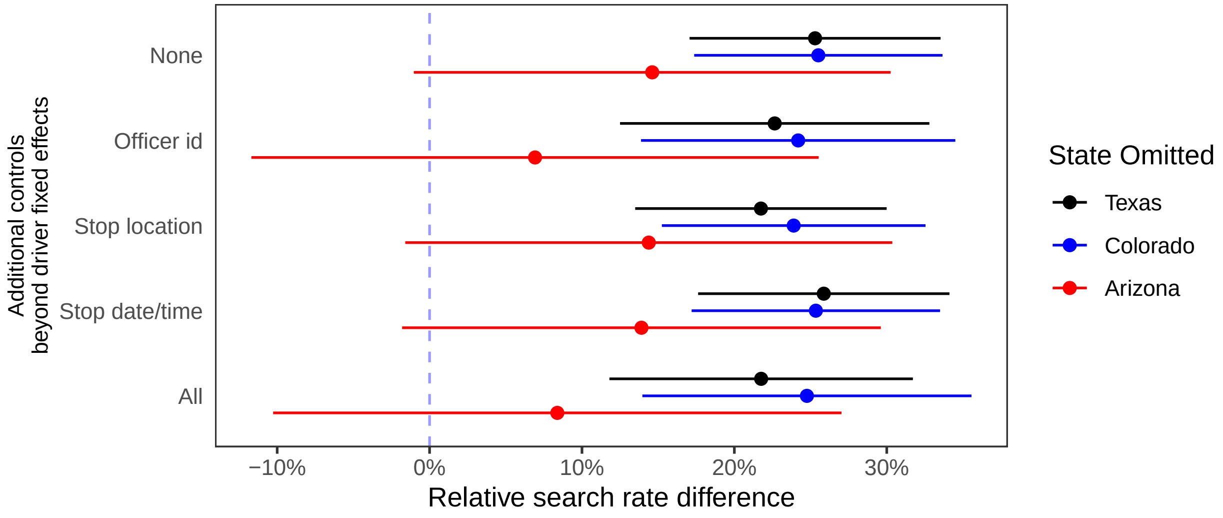

Figure S5: **Estimated relative search difference when omitting one state at a time from the regression, using a linear probability model.** The horizontal axis plots the estimated change in search rate when the same driver is perceived as Hispanic as opposed to white, relative to the overall search rate. (We report estimates relative to the overall search rate to standardize estimates across states, which differ significantly in their search rates.)

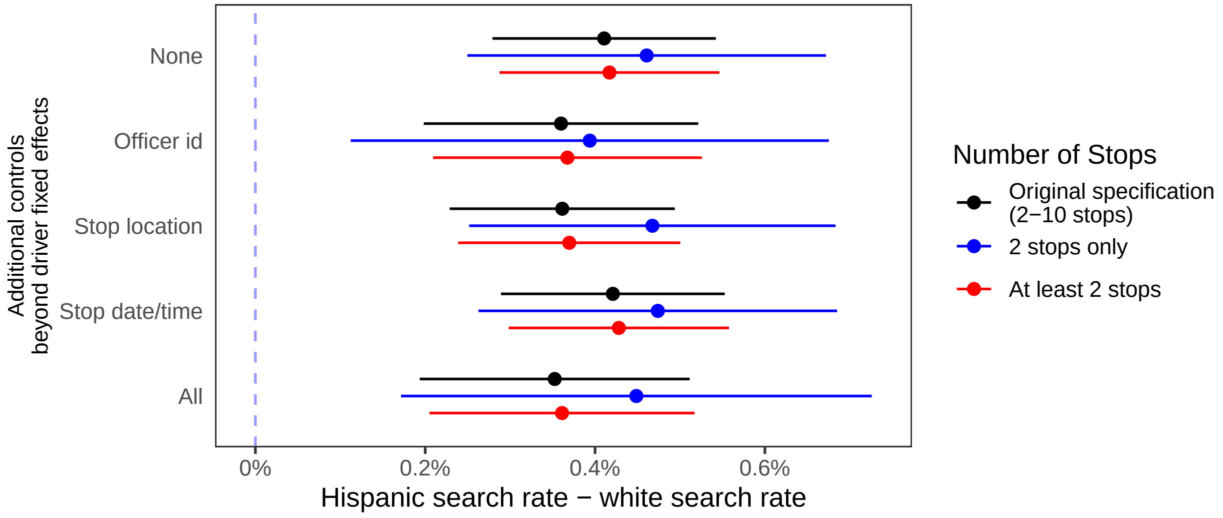

Figure S6: **Estimated increase in the search rate when the same driver is perceived as Hispanic as opposed to white, filtering the data for different numbers of stops per driver.** We report estimates when filtering for drivers with 2-10 stops (as in our original specification; black points); exactly 2 stops (blue points); and at least two stops (red points).

REFERENCES AND NOTES

1. D. Pager, H. Shepherd, The sociology of discrimination: Racial discrimination in employment, housing, credit, and consumer markets. *Annu. Rev. Sociol.* **34**, 181–209 (2008).

2. P. Kline, E. K. Rose, C. R. Walters, Systemic discrimination among large U.S. employers. *Q. J. Econ.* **137**, 1963–2036 (2022).

3. E. Pierson, C. Simoiu, J. Overgoor, S. Corbett-Davies, D. Jenson, A. Shoemaker, V. Ramachandran, P. Barghouty, C. Phillips, R. Shroff, S. Goel, A large-scale analysis of racial disparities in police stops across the United States. *Nat. Hum. Behav.* **4**, 736–745 (2020).

4. C. Simoiu, S. Corbett-Davies, S. Goel, The problem of infra-marginality in outcome tests for discrimination. *Ann. Appl. Stat.* **11**, 1193–1216 (2017).

5. B. Edelman, M. Luca, D. Svirsky, Racial discrimination in the sharing economy: Evidence from a field experiment. *Am. Econ. J. Appl. Econ.* **9**, 1–22 (2017).

6. D. R. Williams, R. Wyatt, Racial bias in health care and health: Challenges and opportunities. *JAMA* **314**, 555–556 (2015).

7. R. Neil, C. Winship, Methodological challenges and opportunities in testing for racial discrimination in policing. *Annu. Rev. Criminol.* **2**, 73–98 (2019).

8. G. Ridgeway, Assessing the effect of race bias in post-traffic stop outcomes using propensity scores. *J. Quant. Criminol.* **22**, 1–29 (2006).

9. J. Knowles, N. Persico, P. Todd, Racial bias in motor vehicle searches: Theory and evidence. *J. Pol. Econ.* **109**, 203–229 (2001).

10. R. Wilms, E. Mäthner, L. Winnen, R. Lanwehr, Omitted variable bias: A threat to estimating causal relationships. *Methods Psychol.* **5**, 100075 (2021).

11. M. Bertrand, S. Mullainathan, Are Emily and Greg more employable than Lakisha and Jamal? A field experiment on labor market discrimination. *Am. Econ. Rev.* **94**, 991–1013 (2004).

12. J. Grogger, G. Ridgeway, Testing for racial profiling in traffic stops from behind a veil of darkness. *J. Am. Stat. Assoc.* **101**, 878–887 (2006).

13. A. Gelman, J. Fagan, A. Kiss, An analysis of the New York City police department's "stop-and-frisk" policy in the context of claims of racial bias. *J. Am. Stat. Assoc.* **102**, 813–823 (2007).

14. G. Ridgeway, J. M. MacDonald, Doubly robust internal benchmarking and false discovery rates for detecting racial bias in police stops. *J. Am. Stat. Assoc.* **104**, 661–668 (2009).

15. D. Arnold, W. Dobbie, P. Hull, Measuring racial discrimination in bail decisions. *Am. Econ. Rev.* **112**, 2992–3038 (2022).

16. D. E. Broockman, E. J. Soltas, A natural experiment on discrimination in elections. *J. Public Econ.* **188**, 104201 (2020).

17. K. L. Milkman, M. Akinola, D. Chugh, Temporal distance and discrimination: An audit study in academia. *Psychol. Sci.* **23**, 710–717 (2012).

18. E. K. Rose, A constructivist perspective on empirical discrimination research. *J. Econ. Lit.* **61**, 906–923 (2023).

19. D. R. Harris, J. J. Sim, Who is Multiracial? Assessing the Complexity of Lived Race. *Am. Sociol. Rev.* **67**, 614–627 (2002).

20. A. Saperstein, A. Gullickson, A "mulatto escape hatch" in the United States? Examining evidence of racial and social mobility during the Jim Crow era. *Demography* **50**, 1921–1942 (2013).

21. A. Saperstein, A. M. Penner, J. M. Kizer, The criminal justice system and the racialization of perceptions. *Ann. Am. Acad. Pol. Soc. Sci.* **651**, 104–121 (2014).

22. R. Dahis, E. Nix, N. Qian, *Choosing racial identity in the United States, 1880–1940* (National Bureau of Economic Research, 2019).

23. H. Noghanibehambari, J. Fletcher, *Passing as White: Racial Identity and Old-Age Longevity* (National Bureau of Economic Research, 2025).

24. J. B. Freeman, A. M. Penner, A. Saperstein, M. Scheutz, N. Ambady, Looking the part: Social status cues shape race perception. *PLOS ONE* **6**, e25107 (2011).

25. G. Nicolas, A. L. Skinner, C. L. Dickter, Other than the sum: Hispanic and Middle Eastern categorizations of Black-white mixed-race faces. *Soc. Psychol. Pers. Sci.* **10**, 532–541 (2018).

26. J. B. Freeman, N. Ambady, A dynamic interactive theory of person construal. *Psychol. Rev.* **118**, 247–279 (2011).

27. C. N. Macrae, G. V. Bodenhausen, Social cognition: Thinking categorically about others. *Annu. Rev. Psychol.* **51**, 93–120 (2000).

28. T. A. Ito, G. R. Urland, Race and gender on the brain: Electrocortical measures of attention to the race and gender of multiply categorizable individuals. *J. Pers. Soc. Psychol.* **85**, 616–626 (2003).

29. J. L. Eberhardt, P. A. Goff, V. J. Purdie, P. G. Davies, Seeing black: Race, crime, and visual processing. *J. Pers. Soc. Psychol.* **87**, 876–893 (2004).

30. O. H. MacLin, R. S. Malpass, Racial categorization of faces: The ambiguous race face effect. *Psychol. Public Policy Law* **7**, 98–118 (2001).

31. B. Duncan, S. J. Trejo, Which Mexicans are white? Enumerator-assigned race in the 1930 census and the socioeconomic integration of mexican americans. *ILR Rev.* **78**, 62–85 (2025).

32. A. Saperstein, Double-checking the race box: Examining inconsistency between survey measures of observed and self-reported race. *Soc. Forces* **85**, 57–74 (2006).

33. M. R. Herman, Do you see what i am?: How observers' backgrounds affect their perceptions of multiracial faces. *Soc. Psychol. Q.* **73**, 58–78 (2010).

34. E. J. Baron, J. J. Doyle Jr, N. Emanuel, P. Hull, J. Ryan, *Unwarranted Disparity in High-Stakes Decisions: Race Measurement and Policy Responses* (National Bureau of Economic Research, 2024).

35. K. Finlay, E. Luh, M. G. Mueller-Smith, *Implications of Race and Ethnicity (mis) measurement in the US Criminal Justice System* (National Bureau of Economic Research, 2024).

36. J. M. Samalik, C. S. Goldberg, Z. J. Modi, E. M. Fredericks, S. K. Gadepalli, S. J. Eder, J. Adler, Discrepancies in race and ethnicity in the electronic health record compared to self-report. *J. Racial Ethn. Health Disparities* **10**, 2670–2675 (2023).

37. W. D. Roth, The multiple dimensions of race. *Ethn. Racial Stud.* **39**, 1310–1338 (2016).

38. M. Sen, O. Wasow, Race as a bundle of sticks: Designs that estimate effects of seemingly immutable characteristics. *Annu. Rev. Polit. Sci.* **19**, 499–522 (2016).

39. A. Laniyonu, S. T. Donahue, Effect of racial misclassification in police data on estimates of racial disparities. *Crim.* **61**, 295–315 (2023).

40. R. Mill, L. C. Stein, Race, skin color, and economic outcomes in early twentieth-century America (Social Science Research Network, 2016).

41. R. Abramitzky, J. Conway, R. Mill, L. Stein, *The Gendered Impacts of Perceived Skin Tone: Evidence from African-American Siblings in 1870–1940*, (National Bureau of Economic Research, 2023).

42. C. Cornwell, J. Rivera, I. M. Schmutte, Wage discrimination when identity is subjective: Evidence from changes in employer-reported race. *J. Hum. Resour.* **52**, 719–755 (2017).

43. S. Goel, J. M. Rao, R. Shroff, Precinct or prejudice? Understanding racial disparities in New York City's stop-and-frisk policy. *Ann. Appl. Stat.* **10**, 365–394 (2016).

44. E. Pierson, S. Corbett-Davies, S. Goel, Fast threshold tests for detecting discrimination, in *International Conference on Artificial Intelligence and Statistics* (PMLR, 2018), pp. 96–105.

45. M. H. Lopez, J. M. Krogstad, J. Passel, Who is Hispanic? *Pew Research Center* (2023).

46. E. P. Monk Jr., Skin tone stratification among Black Americans, 2001–2003. *Soc. Forces* **92**, 1313–1337 (2014).

47. E. P. Monk Jr., The cost of color: Skin color, discrimination, and health among African-Americans. *Am. J. Sociol.* **121**, 396–444 (2015).

48. E. P. Monk, The color of punishment: African Americans, skin tone, and the criminal justice system. *Ethn. Racial Stud.* **42**, 1593–1612 (2019).

49. A. Saperstein, J. M. Kizer, A. M. Penner, Making the most of multiple measures: Disentangling the effects of different dimensions of race in survey research. *Am. Behav. Sci.* **60**, 519–537 (2016).

50. D. D. McAlpine, T. J. Beebe, M. Davern, K. T. Call, Agreement between self-reported and administrative race and ethnicity data among Medicaid enrollees in Minnesota. *Health Serv. Res.* **42**, 2373–2388 (2007).

51. R. Hasnain-Wynia, K. Van Dyke, M. Youdelman, C. Krautkramer, S. L. Ivey, R. Gilchick, E. Kaleba, M. K. Wynia, Barriers to collecting patient race, ethnicity, and primary language data in physician practices: An exploratory study. *J. Natl. Med. Assoc.* **102**, 769–775 (2010).

52. K. Arrow, O. Ashenfelter, A. Rees, Discrimination in labor markets, in *The Theory of Discrimination* (1973), pp. 3–33.

53. S. Starr, Statistical discrimination. *Harv. Civ. Rights-Civ. Liberties Law Rev.* **58**, 579–662 (2023).

54. C. Goldin, C. Rouse, Orchestrating impartiality: The impact of “blind” auditions on female musicians. *Am. Econ. Rev.* **90**, 715–741 (2000).

55. N. Huntington-Klein, *The effect: An introduction to research design and causality* (Chapman and Hall/CRC, 2021); https://theeffectbook.net/index.html.

56. S. R. Porter, C. A. Liebler, J. M. Noon, An outside view: What observers say about others' races and Hispanic origins. *Am. Behav. Sci.* **60**, 465–497 (2016).

57. S. Anwar, H. Fang, An alternative test of racial prejudice in motor vehicle searches: Theory and evidence. *Am. Econ. Rev.* **96**, 127–151 (2006).

58. E. Pierson, C. Simoiu, J. Overgoor, S. Corbett-Davies, V. Ramachandran, C. Phillips, S. Goel, A large-scale analysis of racial disparities in police stops across the United States. arXiv:1706.05678 [stat.AP] (2017).

59. A. Ream, Three decades of multiracial identity research: A bibliometric review. *Identity* **23**, 267–287 (2023).

60. M. Shih, D. T. Sanchez, When race becomes even more complex: Toward understanding the landscape of multiracial identity and experiences. *J. Soc. Issues.* **65**, 1–11 (2009).

61. S. E. Gaither, Mixed results: Multiracial research and identity explorations. *Curr. Dir. Psychol. Sci.* **24**, 114–119 (2015).

62. J. M. Chen, D. L. Hamilton, Natural ambiguities: Racial categorization of multiracial individuals. *J. Exp. Soc. Psychol.* **48**, 152–164 (2012).

63. I. Ayres, Outcome tests of racial disparities in police practices. *Justice Res. Policy* **4**, 131–142 (2002).

64. Y. Irizarry, E. P. Monk Jr., R. J. Cobb, Race-shifting in the United States: Latinxs, skin tone, and ethnoracial alignments. *Sociol. Race Ethn.* **9**, 37–55 (2023).

65. R. D. Alba, S. Lindeman, N. E. Insolera, Is race really so fluid? Revisiting Saperstein and Penner's empirical claims. *Am. J. Sociol.* **122**, 247–262 (2016).

66. N. Vargas, K. Stainback, Documenting contested racial identities among self-identified Latina/os, Asians, Blacks, and Whites. *Am. Behav. Sci.* **60**, 442–464 (2016).

67. D. M. Amodio, The neuroscience of prejudice and stereotyping. *Nat. Rev. Neurosci.* **15**, 670–682 (2014).

68. D. Knox, W. Lowe, J. Mummolo, Administrative records mask racially biased policing. *Am. Polit. Sci. Rev.* **114**, 619–637 (2020).

69. J. Gaebler, W. Cai, G. Basse, R. Shroff, S. Goel, J. Hill, A causal framework for observational studies of discrimination. *Stat. Public Policy* **9**, 26–48 (2022).

70. F. Goncalves, S. Mello, A few bad apples? Racial bias in policing. *Am. Econ. Rev.* **111**, 1406–1441 (2021).

71. J. D. Gaebler, S. Goel, A simple, statistically robust test of discrimination. *Proc. Natl. Acad. Sci. U.S.A.* **122**, e2416348122 (2025).

72. E. Luh, Not so black and white: Uncovering racial bias through systematically misreported trooper reports (SSRN, 2022).

73. B. Friberg, D. Barer, R. Garza, J. Hinkle, R. Sims, C. Bien, P. Tolbert, C. Cross, Texas troopers ticketing Hispanic drivers as white (KXAN News, 2015); https://www.kxan.com/investigations/texas-troopers-ticketing-hispanic-drivers-as-white/.

74. S. Moscou, M. R. Anderson, J. B. Kaplan, L. Valencia, Validity of Racial/Ethnic Classifications in Medical Records Data: An Exploratory Study. *Am. J. Public Health* **93**, 1084–1086 (2003).

75. F. C. G. Polubriaginof, P. Ryan, H. Salmasian, A. W. Shapiro, A. Perotte, M. M. Safford, G. Hripcsak, S. Smith, N. P. Tatonetti, D. K. Vawdrey, Challenges with quality of race and ethnicity data in observational databases. *J. Am. Med. Inform. Assoc.* **26**, 730–736 (2019).

76. N. R. Pettit, K. A. Lane, L. Gibbs, P. Musey, X. Li, J. R. Vest, Concordance Between Electronic Health Record-Recorded Race and Ethnicity and Patient Report in Emergency Department Patients. *Ann. Emerg. Med.* **84**, 111–117 (2024).

77. C. Berry, S. A. Kaplan, T. Mijanovich, A. Mayer, Moving to patient reported collection of race and ethnicity data: Implementation and impact in ten hospitals. *Int. J. Health Care Qual. Assur.* **27**, 271–283 (2014).

78. Z. Obermeyer, B. Powers, C. Vogeli, S. Mullainathan, Dissecting racial bias in an algorithm used to manage the health of populations. *Science* **366**, 447–453 (2019).

79. E. J. Baron, J. J. Doyle Jr., N. Emanuel, P. Hull, J. Ryan, Discrimination in Multiphase Systems: Evidence from Child Protection. *Q. J. Econ.* **139**, 1611–1664 (2024).

80. S. Balachandar, N. Garg, E. Pierson, Domain constraints improve risk prediction when outcome data is missing. arXiv:2312.03878 [cs.LG] (2024).

81. E. Pierson, D. M. Cutler, J. Leskovec, S. Mullainathan, Z. Obermeyer, An algorithmic approach to reducing unexplained pain disparities in underserved populations. *Nat. Med.* **27**, 136–140 (2021).

82. F. R. Baumgartner, B. D. Jones, J. Zaconet, C. Wilson, A. Krishnamurthy, Racial Disparities in Texas Department of Public Safety Traffic Stops (2015), pp. 2002–2014. https://fbaum.unc.edu/TrafficStops/Baumgartner-TexasDPS-Nov2015.pdf.

83. S. Goel, M. Meredith, M. Morse, D. Rothschild, H. Shirani-Mehr, One person, one vote: Estimating the prevalence of double voting in US presidential elections. *Am. Polit. Sci. Rev.* **114**, 456–469 (2020).

84. N. Breslow, N. Day, K. Halvorsen, R. Prentice, C. Sabai, Estimation of multiple relative risk functions in matched case-control studies. *Am. J. Epidemiol.* **108**, 299–307 (1978).
